# Carbon Dioxide Embolism During Transanal Total Mesorectal Excision: Case Report and Literature Review

**DOI:** 10.3389/fsurg.2022.873964

**Published:** 2022-05-06

**Authors:** Youzhuang Zhu, Weiwei Wang, Dingsheng Liu, Hong Zhang, Lina Chen, Zhichao Li, Shangyuan Qin, Yihan Kang, Jun Chai

**Affiliations:** ^1^ Department of Anesthesiology, The Affiliated Hospital of Qingdao University, Qingdao, China; ^2^ Department of Anesthesiology, Weihai Municipal Hospital, Cheeloo Colledge of Medicine, Shandong University, Weihai, China; ^3^ Department of Colorectal Tumor Surgery, Shengjing Hospital of China Medical University, Shenyang, China; ^4^ Department of Anesthesiology, Shandong Provincial Qianfoshan Hospital, Jinan, China; ^5^ Department of Anesthesiology, Shengjing Hospital of China Medical University, Shenyang, China

**Keywords:** carbon dioxide embolism, transanal total mesorectal excision, etiology, prevention, case report

## Abstract

The actual incidence of carbon dioxide embolism during transanal total mesorectal excision (taTME) is unknown, but the reported incidence in the existing literature is reassuring. However, the incidence of CO_2_ embolism, which can be life-threatening, is severely underestimated. By reviewing the available data on carbon dioxide embolism during taTME and synthesizing other reports on CO_2_ embolism in laparoscopic procedures, we provide the first comprehensive account of the etiology, pathophysiology, and recommend tools to monitor carbon dioxide embolism during taTME. Additionally, we provide guidance and recommendations on preventive and therapeutic measures to minimize the adverse consequences of this potentially severe complication, knowledge about which we hope will improve patients’ safety.

## Introduction

Transanal total mesorectal excision (taTME) was introduced in 2010 to improve oncologic, functional, and early post-operative results in carcinoma located in the lower and middle third of the rectum (1). TaTME is fundamentally different from conventional laparoscopic surgery. TaTME is insufflation of carbon dioxide (CO_2_) in a potential space created by incremental dissection to achieve exposure and identification of the correct tissue plane, whereas conventional laparoscopy is performed in a real space that is constant and need not be created by dissection (2). Moreover, whereas conventional laparoscopic surgery requires only transabdominal insufflation of CO_2_, taTME often requires both transanal and transabdominal insufflation of CO_2_. Therefore, their Newtonian fluid dynamics and gas dynamics are different (2).

With increasing popularity of this approach, related complications have attracted much attention. Among the many potential risks, CO_2_ embolism is a rare but potentially fatal complication (3). Research indicates that the risk of CO_2_ embolism in taTME is moderate, whereas in conventional laparoscopic surgery it is extremely low (2). At our surgical treatment center, a 58-year-old male underwent taTME for rectal cancer. He suffered a cardiac arrest secondary to CO_2_ embolism during the procedure. After deflation of the body cavity and resuscitation, he survived. The differences between insufflation in taTME and laparoscopy prompted a literature review to identify causative factors and preventive measures for this rare complication. Unfortunately, only case reports of CO_2_ embolism during taTME (4–7) but no detailed description of its mechanism, pathophysiology, and preventive measures were available. The aim of this case report and review is to provide a basis for future guidelines on prevention and treatment of this severe complication.

## Case Presentation

We report a case of rectal cancer in a 50-year-old man who was scheduled for taTME under general anesthesia. The lower edge of the tumor was 3 cm from the dentate line. The patient had no history of hypertension, coronary heart disease, diabetes, cerebrovascular disease, or pneumonia. Blood routine, blood biochemistry, electrocardiogram, heart color doppler ultrasound, chest radiography, and lung function examinations before surgery showed no obvious abnormalities.

**Figure 1:**
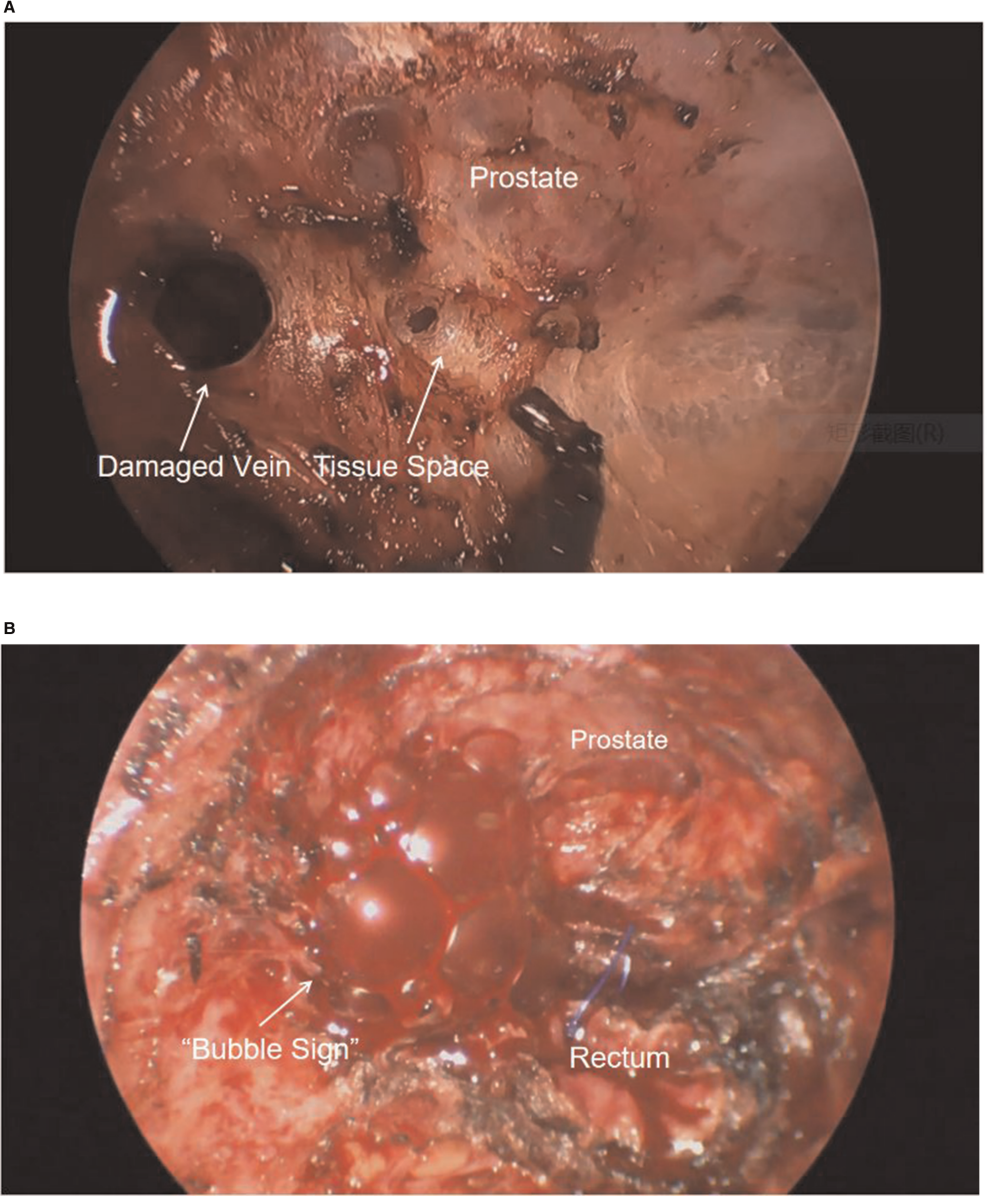
(**A**) Damaged veins remain open under the pneumopelvis. (**B**) “Bubble sign” after carbon dioxide enters the damaged vein.

After general anesthesia, the radial artery was punctured and connected to a manometry kit, the right internal jugular vein was punctured, and a central venous catheter was placed to a depth of 14 cm. The patient was placed in a Trendelenburg position, which granted access for both abdominal and transanal teams to operate simultaneously. Abdominal pneumoperitoneum pressure was adjusted to 13 mmHg. The transanal team used a Lonestar retractor to hold the anus open, and positioned the Starport single-hole device. They performed purse-string suture at a distance of 1 cm from the lower edge of the tumor under the endoscope, and then incised the full thickness of the rectal wall 1 cm below the purse to dissociate from bottom to top. The transanal team used a standard insufflator device, and adjusted the initial pneumopelvic pressure to 13 mmHg. They noticed minimal bleeding on the surface of the prostate, while using electrocautery to separate the space anterior to the rectum. However, the smoke produced forced the surgeon to use a suction device to continue aspiration during the procedure. To offset the loss of pneumopelvic pressure due to smoke aspiration, they had to set the pneumopelvic pressure to 15 mmHg. A video review of the surgery revealed that a venous breach was present on the surface of the prostate at this time. Due to the pneumopelvic pressure, the breach did not bleed but rather changed into a circular cavity (Figure 1A). However, the surgeons misconstrued it as a tissue space.

The anesthesiologist found that the end-expiratory carbon dioxide pressure (P_ET_CO_2_) gradually increased from 41 mmHg to 61 mmHg in 30 min. The team quickly performed the first arterial blood gas analysis, which detected elevated arterial carbon dioxide pressure and respiratory acidosis (PH: 7.147, PaCO_2_: 86 mmHg, $\mathrm{HC}O_{3}{}^{-}$: 30.9 mmHg, Lac: 1.2 mmol/L, BE: –0.21 mmol/L). The anesthesiologist immediately advised the surgeon to suspend the pneumoperitoneum and perform pure oxygen hyperventilation to expedite the elimination of CO_2_. The anesthesiologist again advised the surgeon to suspend the pneumopelvis due to the insignificant decrease in P_ET_CO_2_. After the suspension of the pneumopelvis, the surgeon noticed increased bleeding from the transanal free area and immediately applied gauze compression to stop the bleeding. After a 10-minute suspension of the pneumoperitoneum and pneumopelvis, P_ET_CO_2_ dropped to 40 mmHg, and the transanal team re-established the pneumopelvis. After the pneumopelvis was established, the transanal team noticed a significant reduction in bleeding through endoscopic exploration and sought to continue the surgery. However, the anesthesiologist noted a sudden decrease in P_ET_CO_2_ to 5 mmHg, a blood oxygen saturation decrease from 97% to 91%, invasive blood pressure from 125/85 mmHg to 70/33 mmHg, and heart rate from 70 to 42 bpm. Suspecting CO_2_ embolism, the anesthesiologists immediately injected ephedrine 12 mg intravenously for circulatory support, while the surgeon deflated the perineal cavity and discontinued the surgery. During the pneumopelvic deflation, several gas bubbles appeared in the vascular breach (Figure 1B).

Unfortunately, the patient's hemodynamics deteriorated further, manifesting as circulatory collapse (blood pressure fell to 43/20 mmHg and heart rate fell to 26 bpm). The surgeon immediately initiated external chest compressions, and the anesthesiologist administered 1 mg of epinephrine through a central venous catheter for circulatory support. A total of 4 mg of epinephrine was administered over the four-minute period in which circulatory collapse occurred. The use of epinephrine and continued chest compressions did not improve circulatory collapse, as manifested by persistent mean arterial pressure below 50 mmHg. The second blood gas analysis detected severe expiratory acidosis, compensated alkalosis, and metabolic acidosis (PH: 7.255, PaCO_2_: 117.2 mmHg, $\mathrm{HC}O_{3}{}^{-}$: 50.9 mmHg, Lac: 6.6 mmol/L, BE: 18.23 mmol/L). The anesthesiologist used an ice cap to implement controlled hypothermia to reduce the metabolism of brain tissue, and 100 mL mannitol was used to prevent cerebral edema due to cerebral hypoperfusion. Furthermore, the anesthesiologist tried unsuccessfully to extract air through the central catheter. The patient soon developed ventricular fibrillation, and the anesthesiologist immediately administered defibrillation (non-synchronized, 200 J). Epinephrine (1 mg) was again administered through the central venous catheter. Two minutes later, the patient had relapsed into ventricular fibrillation and the anesthesiologist repeated electrical defibrillation (non-synchronized, 200 J). Fortunately, the patient regained sinus rhythm after the second defibrillation, but the hypotension persisted. To maintain hemodynamic stability, the patient received a continuous intravenous infusion of norepinephrine (initial rate 0.05 µg/kg/min, maximum rate 0.1 µg/kg/min). The patient's invasive arterial pressure gradually increased, and the heart rate and P_ET_CO_2_ gradually improved. Thirty minutes later, the anesthesiologist evaluated the patient and confirmed that the vital signs and cardiopulmonary function were stable. The third arterial blood gas analysis detected an attenuated expiratory acidosis and metabolic acidosis (PH: 7.238, PaCO_2_: 54.6 mmHg, $\mathrm{HC}O_{3}{}^{-}$: 22.8 mmHg, Lac: 6.1 mmol/L, BE: –5.19 mmol/L). The surgeons stopped the endoscopy and converted to transabdominal open surgery, followed by ultra-low anterior resection anastomosis and ileal protective stoma. The bleeding vessel was found to be the superficial vein of prostate. After surgery, the patient was transferred to the intensive care unit with endotracheal intubation. The surgery was of 5 h duration, and the intraoperative blood loss was 200 mL. On day 3 post-surgery, the patient's cardiopulmonary function was stable, and the patient underwent contrast-enhanced CT of the chest, abdomen, and lower extremities to confirm the absence of pulmonary embolism or thrombosis before being transferred back to the general surgery ward. After ventilation therapy was discontinued, the patient had transient nervous system symptoms, such as hallucinations and agitation. These symptoms were relieved after symptomatic treatment, such as neurotrophic therapy for two days. Since then, no neurological sequelae have been recorded seven months post-surgery.

In this case, the diagnosis of CO_2_ embolism was based on a rapid decrease in P_ET_CO_2_, sharp deterioration in hemodynamics and arterial blood gas analysis, and venous rupture on review of imaging data. Unfortunately, our anesthesia center does not have equipment, such as transesophageal echocardiography and precordial Doppler, to provide evidence on imaging. However, the striking clinical signs and the patient's rapid postoperative recovery give sufficient reason to believe that the patient experienced severe CO_2_ embolism rather than a cardiac or other disease.

## Literature Review

In recent years, taTME has become the focus of surgical treatment of low rectal cancer. In 2013, Rouanet et al. (3) first reported CO_2_ embolism in taTME. From 2016 to 2019, four cases of CO_2_ embolism during taTME were reported (4–7). According to Harnsberger et al. (5), CO_2_ embolism occurred in three (3.8%) of 80 taTME surgeries. In 2019, data from the LOREC and OSTRiCH registries (8) showed that 25 (0.4%) patients of 6,375 patients who underwent taTME had CO_2_ embolism. However, Lanier et al. (9) noted that the true incidence of occult CO_2_ embolism during taTME may be underestimated. We reviewed these studies and summarized the clinical characteristics of patients with CO_2_ embolism during taTME (Table 1).

**Table 1:** Clinical case reports of CO_2_ embolism during taTME.

Study	Gender (n)	Diagnosis (n)	Operation	Position (n)	Source (n)	Location (n)	Events	Outcome
1 (3)	M	Rectal cancer	Transanal Endoscopic Proctectomy	A	NA	Posterior prostate resection	NA	NO mortality occur
2 (4)	W	Low rectal cancer	taTME	C	NA	Transanal phase	P_ET_CO_2 _= 13 mmHg BP = 68/45 mmHg SPO_2_ < 75%	Uneventful recovery
3 (5)	M	Rectal Cancer	Laparoscopic LAR	B	a	Transanal phase	P_ET_CO2 = 18 mmHg BP = 90/70 mmHg SPO_2_ <85%	Recovery
	F	Rectal Cancer	Laparoscopic LAR	B	a	Transanal phase	P_ET_CO_2 _= NA BP = 80/58 mmHg SPO_2 _= NA	Recovery
	F	Pelvic abscess Rectovaginal fistula	Laparoscopic LAR	B	a	Transanal phase	P_ET_CO_2 _= 25 mmHg BP = 80/50 mmHg SPO_2 _= 89%	Recovery
4 (6)	M	Low rectal cancer	taTME	B	a	Dissection the prostate	P_ET_CO_2 _= 29 mmHg BP = 135/35 mmHg SPO_2 _= 94%	Vital signs did not worsen
	M	Recurrence of rectal cancer	taTME	B	a	Dissection of the posterior	P_ET_CO_2 _= 16 mmHg BP = 67/57 mmHg SPO_2 _= 97%	Vital signs recovery
5 (7)	M	Anorectal adenocarcinoma	Transperineal approach in total pelvic exenteration	A	b	Transanal phase	P_ET_CO_2 _= 14 mmHg BP = 70/40 mmHg SPO_2 _= 68%	Vital signs recovery
6 (8)	M (19) F (6)	Cancer (20) benign (5)	taTME	B (23) D (1)	a (10) b (3) c (5) d (2) e (1) f (4)	Transanal phase (10) abdominal and transanal (15)	P_ET_CO_2_ reduction by >30% BP = NA SPO_2 _ < 92%	No deaths occurred

*A, lithotomy position; B, Trendelenburg position; C, modified Lloyd-Davies position with Trendelenburg tilt; D, flat; NA, not mentioned in the text; taTME, transanal total mesorectal excision; LAR, low anterior resection; a, periprostatic vein; b, paravaginal veins; c, lateral pelvic vein; d, posterior pelvic vein; e, inferior mesenteric artery; f, no bleeding identified; M, male; F, female; BP, blood pressure; SPO_2_, peripheral capillary oxygen saturation; P_ET_CO_2_, end-tidal carbon dioxide partial pressure*.

### Etiology of Carbon Dioxide Embolism during taTME

The etiology of CO_2_ embolism in taTME is multifactorial, but in this review we summarize several plausible contributing factors.

#### The Insufflation of CO_2_ in the taTME Produces Certain Characteristic Effects as Follows

(a) anatomical distortions (2). During taTME, insufflation of CO_2_ into the pelvic space is required to create the operating space, which places significant forward pressure on the rectum and its mesentery, resulting in deformation of the anatomy. Surgeons tend to injure the high-velocity prostatic or paravaginal venous plexus in the wrong anatomical plane. The prostate is rich in blood supply, surrounded by venous plexuses and bilateral neurovascular bundles. This may also explain why 19 of the 25 patients with CO_2_ embolism reported in the 2019 International Registry (8) were male. For women, the rich blood supply of the vaginal wall and surrounding venous plexuses lead to an increased risk of embolism after bleeding. (b) Damaged blood vessels remain open (10). The partially damaged blood vessel cannot be closed under the action of pneumopelvic pressure, and the open state of the vascular lumen allows CO_2_ to enter the circulation continuously. In contrast, if blood vessels of the same caliber are completely transected, the blood vessel cavity will collapse under the action of pneumopelvic pressure. (c) Cyclic billowing (2), defined as the sudden and repetitive collapse of the operative work space caused by pneumatic instability, is characteristic of taTME. It is the most common technical complication during taTME in the largest registry data series published to date (11). Insufflation mode and continuum mechanics of gas delivery are major causes of cyclic billowing. AirSeaL® is a CO_2_ insufflation system most used by taTME surgeons. It can respond immediately to small changes in pressure and provide a more stable pneumatic work pace by independently regulating CO_2_ pressure, which minimizes cycle billowing (2). However, most units performing taTME procedures in our country are not equipped with the AirSeal® system. Without the AirSeal® system, surgeons had to continuously pump smoke with a suction device to clear the surgical field during the procedure. To counteract this loss of pressure, the surgeon will set the pneumopelvic pressure and flow rate at higher levels, which increases the incidence of CO_2_ embolism.

#### Pressure Gradient Facilitates CO_2_ Entry into Systemic Circulation

(a) With standard insufflators, surgeons often increase the pressure (15–17 mmHg) on the transanal insufflation device to obtain the best working field of view (12). The limited pelvic space further increases pneumopelvic pressure, creating a pressure gradient with the lower colorectal venous plexus that entrains CO_2_ into circulation. (b) During taTME, the patient is often placed in the Trendelenburg position (5). The venous pressure in the pelvic veins is decreased in this position since the surgical field is above the level of the heart, creating a larger pressure gradient during CO_2_ insufflation at 15–17 mmHg. One study (13) revealed that a pressure gradient of 5 mmHg is sufficient to allow entry of CO_2_ into the venous system, and the degree of the Trendelenburg position was directly related to the rate of CO_2_ embolism.

### Pathophysiology of Carbon Dioxide Embolism during taTME

It is known that CO_2_ entering the circulatory system produces only weak clinical changes in most cases, but can be fatal in some extreme cases. This clinical range is due to the amount and speed of CO_2_ entrainment, and the presence of paradoxical embolism (14).

In taTME, CO_2_ is slowly and continuously entrained through damaged blood vessels. In the early stages, this mechanism often results in less profound clinical changes due to the pulmonary circulation’s ability to filter small amounts of CO_2_, which diffuses into the alveoli and is exhaled (15). If the damaged blood vessels are not closed in time, CO_2_ will continue to enter the circulatory system, and a large amount of CO_2_ entrainment will eventually exceed the compensatory function of the lungs. A large amount of CO_2_ emboli lodged in the right ventricle or entering the pulmonary artery may result in right ventricular outflow tract obstruction and pulmonary hypertension (16). Subsequently, pulmonary venous return, left ventricular preload, and cardiac output all decrease. The patient's clinical presentation is severe hypotension, hypoxia, arrhythmia and even cardiac arrest. In addition, the process of reconstructing pneumoperitoneum and pneumopelvis needs more attention, at this time, massive and rapid entrainment of CO_2_ will occur simultaneously. The existence of this condition has been confirmed in our case report, but understanding of the amount and speed of gas lethal to humans is limited because only animal data are available (17, 18).

Severe complications from CO_2_ embolism are not entirely dependent on the amount and speed of gas entering the circulation. Even minimal amounts of CO_2_ can have catastrophic consequences. A paradoxical embolism due to a defect in the patient's intracardiac or pulmonary region can occur in the coronary or cerebral circulation (19) and may cause cardiac arrest, malignant arrhythmias, central, and other unpredictable problems. This phenomenon was clearly observed in one patient reported by Dickson et al. (8).

Imbalances in ventilation-perfusion (V_A_/Q) are the main cause of gas exchange abnormalities associated with CO_2_ embolism. Multiple studies of inert gas elimination techniques (20) have demonstrated a predominance of high V_A_/Q areas (which retain a measurable level of perfusion) in the lung, rather than large increases in dead space (regions with ventilation but no perfusion). This means that pure oxygen ventilation during CO_2_ embolism can maintain the patient's oxygen saturation within a certain range without causing severe hypoxia. Therefore, it should be emphasized that hemodynamic instability or even heart failure caused by the above three factors is the main cause of death in patients, rather than hypoxia.

### Monitors of Carbon Dioxide Embolism During taTME

Transesophageal echocardiography (TEE) is the most sensitive method for monitoring gas embolism during laparoscopic surgery, and can detect venous gas embolism as small as 0.02 mL/kg (21). In addition, TEE can effectively detect paradoxical embolism caused by intracardiac defects. Lanier et al. (9) noted that despite its invasiveness, high cost and lack of alarm device, TEE is the preferred monitoring tool in taTME for high-risk patients because it can both quantify and locate air embolism.

The precordial Doppler (PCD) is completely non-invasive, and it can detect venous gas embolism as small as 0.05 mL/kg, so it is almost as sensitive as TEE (21). The Doppler frequency change caused by an air embolism is described as producing a “washing machine” or “drum”-like sound that is easily detected. However, the position and direction of the probe are critical. It is recommended to place the probe over the right heart along the right sternal border to improve the sensitivity with which air entering the pulmonary circulation is detected (22). Souders et al. (20) noted that PCD is the most widely used monitoring method when there is a high risk of intraoperative air embolism.

The pulmonary arterial (PA) catheterization has a lower threshold volume detection (0.25 mL/kg) for air embolism than PCD (21). PA catheterization is highly invasive and is clearly not suitable for routine detection of air embolism since better and less invasive methods exist. Therefore, the use of PA catheters is limited to those patients with significant comorbidities who may benefit from its use as a cardiac output or mixed venous saturation monitoring tool.

The end-expiratory carbon dioxide (P_ET_CO_2_) monitoring is an intraoperative standard and is the most convenient and widely used method for monitoring air embolism, especially during surgical procedures where air embolism is not expected (20). Its sensitivity is comparable to that of a PA catheter. In the present case, we clearly recorded a bidirectional change in P_ET_CO_2_ which initially increased then decreased. The P_ET_CO_2_ increases during the early stages of embolism, as CO_2_ flows into the blood and is released through the lung. However, P_ET_CO_2_ decreases at the later stage, as cardiac output decreases and physiological dead space increases with time. Decreased P_ET_CO_2_ is the earliest clinical symptom reported in many cases, perhaps due to failure by the anesthesiologist to detect CO_2_ embolism at an early stage. Alternatively, P_ET_CO_2_ monitors may have failed to collect data in a timely manner, because the interval used by anesthesia data acquisition systems is arbitrary.

The decrease in blood pressure, and pulse oxygen saturation results from the pathophysiological changes induced by air embolism, and imply that the air embolism has entered an advanced stage. Therefore, they do not offer high sensitivity in detection (21). Multi-slice computed tomography provides high spatial resolution images for detailed assessment of vasculature and cardiac structures at rest and is a diagnostic method for paradoxical embolism (19).

In conclusion, continuous P_ET_CO_2_ analysis and PCD sonography appear to be clinically applicable for patients undergoing taTME, and in most patients, arterial cannulation and invasive blood pressure monitoring are also recommended. In high-risk patients, TEE appears to be the preferred method for monitoring because it is the most sensitive, and it is unique in that it can both quantify the amount of CO_2_ embolism and identify its location. It is important to emphasize that clinical suspicion based on biphasic changes in P_ET_CO_2_ is particularly important in the early stages of CO_2_ embolism. When other more sensitive tools are not available, early identification of P_ET_CO_2_ changes can help the anesthesiologist to intervene as quickly as possible, which can often prevent further deterioration.

### Treatment of Carbon Dioxide Embolism during taTME

**Figure 2:**
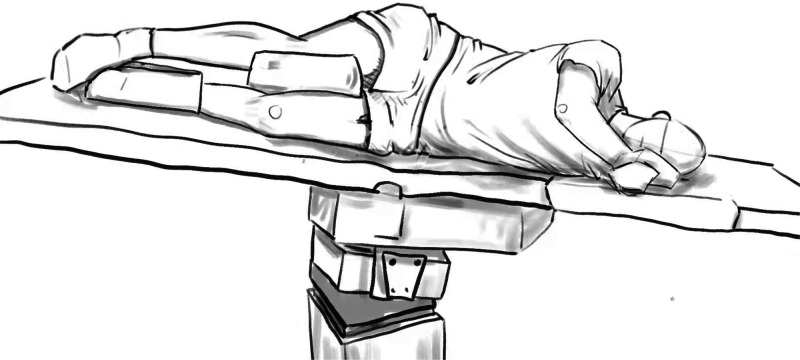
Durant’s position (left side down with head-down); This picture is hand drawn by the authors.

CO_2_ embolism during taTME should be considered when there is a rapid decrease in P_ET_CO_2_, hemodynamic instability, and hypoxemia. At this point, surgery should be suspended. Anesthesiologists and surgeons should immediately take action to prevent further entrainment of CO_2_ and to stabilize the patient. Based on etiology, CO_2_ embolism during taTME may be mainly prevented by the following: (a) The pneumoperitoneum and pneumopelvis need to be released to prevent further embolism. Thereafter, the patient should be ventilated with 100% oxygen to wash out CO_2_ and improve the ventilation perfusion mismatch and hypoxemia (23). (b) The patient should be quickly placed in Durant's position (Figure 2). Due to the inherent buoyancy of CO_2_ embolism, Durant's position allows the CO_2_ embolus to enter the right atrium from the right ventricular outflow tract (24). In addition, estimation of the possibility of an arterial CO_2_ embolism is necessary. In the case of arterial CO_2_ embolism, the patient needs to be placed in a supine position, to avoid Durant's maneuver causing or increasing brain edema (25). (c) Fluid resuscitation will increase central venous pressure, thereby preventing further entry of CO_2_ into the venous circulation (23). Additionally, inotropes, such as ephedrine, phenylephrine, and dopamine, should be administered immediately at the onset of symptoms (23). If a patient’s cardiovascular collapse cannot be corrected and the patient does not respond to adjuvant therapy, cardiopulmonary bypass should be performed (23). (d) If conditions permit, the anesthesiologist should attempt extraction of gas through a central venous catheter (16). Success depends on the patient's position and the position and diameter of the catheter. By using a porous central venous catheter, the catheter opening will be placed into the right atrium or ventricle, which may be of greater clinical significance. (e) Hyperbaric oxygen therapy can prevent neurological deficits after CO_2_ embolism by reducing bubble volume and intracranial pressure, restoring cerebral blood flow and increasing tissue oxygenation (16). However, the clinical effect needs to be further studied, and complications of hyperbaric oxygen therapy should be avoided. When the cause is removed, damaged blood vessels are closed, and vital signs have recovered, continued surgery can be considered. However, it is necessary to closely monitor the prognosis of important organs such as the heart, lungs and brain after surgery. There is no consensus on whether to continue with taTME or switch to open surgery. However, the choices made by the practitioner should not add any further risks to the patient.

### Prevention of CO_2_ Embolism is Particularly Important during taTME

Based on etiology, CO_2_ embolism during taTME may be mainly prevented by following these guidelines: (a) The taTME procedure must be performed by an experienced and skilled surgeon who can recognize the anatomical distortions due to a pneumopelvis (2). When freeing the rectum and its mesentery, the surgeon should try to avoid injury to the prostatic venous plexus or paravaginal venous plexus. (b) When using standard insufflators, in areas where the vascular system is damaged, the “cyclic billowing” caused by repeated suction may prompt the surgeon to set pneumopelvic pressures and flow rates at higher levels to counteract this loss of pressure. This increases the risk of CO_2_ embolism. Therefore, the AirSeaL® systems should be recommended for use in taTME (12). Incorporating basic surgical principles, Bolshinsky et al. (10) advocated the application of pressure with gauze to aid hemostasis, while avoiding the use of suction and potentially reducing the risk of CO_2_ embolism. (c) Pressure from the pneumopelvis exerted on the open veins can mask bleeding, affecting the surgeon’s recognition of injury to small pelvic veins, thereby delaying vessel closure and allowing CO_2_ entry. Therefore, Liu et al. (12) recommended that surgeons immediately identify the exposed periprostatic and paravaginal vessels and quickly close the vessels with either cautery or suture ligation. (d) A decrease in pneumopelvic pressure (10–12 mmHg) reduces the pressure gradient between the pneumopelvis and the colorectal venous plexus (5). Notably, the patient should not be placed in the Trendelenburg position for a sustained period; pressure gradient caused by the surgical site being above the level of the heart tends to increase the risk of CO_2_ embolism (7). If the patient does not have myocardial compromise, crystalloids must be administered preoperatively to increase the central venous pressure (26). (e) Re-insufflation of CO_2_ is a strong risk factor for CO_2_ embolism after deflation (27). Seong et al. (27) therefore recommended avoiding re-establishment of the pneumopelvic before ensuring that the sutured vein is closed during taTME, and paying closer attention to the patient. (f) The presence of a positive pressure gradient between the surgical site and the right atrium is a risk factor for air embolism. Positive end-expiratory pressure (PEEP) can increase right atrial pressure through cardiopulmonary interaction, so the application of PEEP in high-risk surgery (sitting neurosurgery, cesarean section and spine surgery) can reduce the risk of air embolism (20, 28). There also appears to be controversy regarding the use of PEEP for the prevention of air embolism (29, 30). More importantly, however, when large amounts of CO_2_ lead to right ventricular insufficiency or even failure, PEEP should be avoided because it increases right ventricular afterload and reduces cardiac output. (g) The time interval of the data acquisition system should be shortened to detect bidirectional changes in P_ET_CO_2_. The anesthesiologist should set the threshold for P_ET_CO_2_ within a narrow range, and the possibility of CO_2_ embolism should always be considered when biphasic evolution of P_ET_CO_2_ is noticed and a sudden deterioration in hemodynamics occurs. For high-risk patients, TEE or PCD are necessary monitoring methods (7).

## Conclusion

We report a case of CO_2_ embolism during taTME and review the literature to comprehensively describe the etiology, pathophysiology, diagnostic methods, treatment, and prevention of CO_2_ embolism during taTME. Accidental vascular injury caused by anatomical distortion, partially damaged vessels remaining open under the pneumopelvis, and cyclic billowing are unique etiologies of taTME CO_2_ embolism.

The amount and speed of CO_2_ entering the circulation, and the presence or absence of paradoxical embolism determine the pathophysiological evolution of the patient. Continuous P_ET_CO_2_ analysis and PCD sonography appear to be sensible clinical approaches for patients undergoing taTME. In high-risk patients, TEE appears to be the preferred method for monitoring because it is the most sensitive, and it is unique in that it can both quantify the amount of CO_2_ embolism and identify its location. Bidirectional changes in P_ET_CO_2_ require most attention from the anesthesiologist, as they may indicate an imminent CO_2_ embolism. Experienced taTME surgeons, use of the AirSeaL® system, timely identification of damaged blood vessels for treatment, reduction of pneumopelvic pressure, preventive use of PEEP and adjustment of lower limit alarm for P_ET_CO_2_ to a narrower range are important measures for the prevention of taTME CO_2_ embolism. Once CO_2_ embolism occurs, accelerated fluid replacement and vasoactive drug use are needed to maintain stable hemodynamics. Additionally, pure oxygen hyperventilation is needed to correct hypoxemia and hypercapnia. Durant’s position and hyperbaric oxygen therapy may also benefit patients. CO_2_ embolism during taTME is rare and has severe complications. We hope that increased understanding of its mechanism and relevant preventive measures will ensure safety of patients’ surgery and reduce the possibility of CO_2_ embolism.

## Data Availability

The original contributions presented in the study are included in the article/Supplementary Material, further inquiries can be directed to the corresponding author/s.
